# Heart Disease in the Horse

**Published:** 1890-03

**Authors:** John A. McLaughlin

**Affiliations:** Providence, R. I.


### HEART DISEASE IN THE HORSE.

By John A. McLaughlin, D.V.S.
Providence, R. I.

Whether heart disease is as uncommon in the horse as it is generally supposed to be, is questionable to my mind. I believe it to be far more common than the diagnosis. The following case may be of interest to the readers of the Journal.

The patient was a strong built grey gelding and was not suspected by its owner of having any trouble during the time he owned him, a period of about three months.

The history of the case, as given by Mr. Charles Siegel, his owner, was that the animal showed symptoms which he mistook for colic, and although he appeared all right the next day he thought it best to bring the animal to my office ; and while coming here he showed the following symptoms : Heavy, quick, labored breathing, and a cough which obliged Mr. Siegel to take him home without reaching my office.

Shortly after he arrived home I found the temperature 104½°. Of the pulse and respirations I speak from memory. The former was about 40, decidedly weak ; the respirations about 70. This was on the 25th day of July, 1889.

26th. Temperature in morning 105¾° ; in the afternoon it was 105° ; respirations 68 ; pulse 48.

The peculiar weakness of the pulse, and its almost normal number of beats, aroused my suspicions regarding the heart.

27th. Temperature 105¼°; pulse 60; respirations 66.

28th. Temperature 102°; respirations 48; pulse 36. Apparently the animal was doing nobly.

That evening he commenced a series of peculiar symptoms, which returned every twenty-four hours, usually about the same time, until near the end, when they became more frequent. The owner described them as commencing with a trembling. He would then get down hurriedly to save himself from falling, and stretch himself out full length. His respirations would be extremely rapid, quicker than a dog's after running, and he would moan. These symptoms would last about half an hour, when he would get up and be as well as previous to the attack.

The day before the animal died I was fortunate enough to see him in one of the spells. Heretofore I had only seen him after he had recovered, but even then the symptoms were sufficient to base the diagnosis of heart failure. The horse was down; no struggling. For a short time he moved his fore limbs and head, but it was very little; then he stretched himself out full length, his head and neck stretched to their fullest extent, and in that position he remained, without moving, for at least twenty minutes, and with each respiration giving vent to an exaggerated moan or sigh. Part of this time he was unconscious. Of this I took particular notice. His breathing was very difficult, like an animal dying by asphyxia; not breathing very rapidly, but the act of expiration was done by a quick short contraction at the flanks. The pulse was small, fluttering, and irregular; at times it would disappear entirely for several seconds. When the spell passed he quietly got on his feet, looking a very sick horse to be sure, but certainly giving no evidence of having gone through such an uncommon experience.

Post-mortem, held in presence of Drs. Eddy and Farrell, showed hypertrophy of the heart, fatty degeneration, pericarditis and endocarditis and disease of the valves. The valves were very delicate, and the chordæ tendineæ were very frail and attenuated.

